# Supplementary material for: DeepBiomarker: Identifying Important Lab Tests from Electronic Medical Records for the Prediction of Suicide-Related Events among PTSD Patients
Source: J Pers Med. 2022 Mar 24;12(4):524. doi: 10.3390/jpm12040524 (PMC9025406; doi:10.3390/jpm12040524)
Supplement: Supplementary file 1 [file jpm-12-00524-s001.zip › jpm-1619967-supplementary.pdf]

Table S1: Important features identified by perturbation-based contribution analysis for SRE prediction:

**Feature Name**	**Diagnosis Code/DrugBankID/Lab Test**	**Relative Contribution**	**CI95up**	**CI95down**	**FDR_Q**	**p_bonferroni**
Esophageal reflux	530.81	1.72	1.79	1.65	1.10×10^{-128}	2.20×10^{-128}
EPINASTINE HCL 0.05% EYE DROPS	DB00751	2.76	3.02	2.53	6.09×10^{-99}	1.83×10^{-98}
Major depressive disorder, single episode, unspecified	F32.9	1.58	1.65	1.52	6.99×10^{-87}	2.80×10^{-86}
Nicotine dependence, cigarettes, uncomplicated	F17.210	1.52	1.58	1.46	1.58×10^{-80}	7.90×10^{-80}
Personal history of transient ischemic attack (TIA), and cerebral infarction without residual deficits	V12.54	1.82	1.93	1.72	2.21×10^{-77}	1.33×10^{-76}
Anxiety state, unspecified	300	1.50	1.56	1.44	7.55×10^{-74}	5.29×10^{-73}
GLUCOSE	GLUCOSE	1.45	1.51	1.38	9.19×10^{-52}	7.35×10^{-51}
Periumbilical pain	R10.33	1.88	2.04	1.74	5.25×10^{-49}	4.72×10^{-48}
Bipolar disorder, unspecified	F31.9	1.74	1.86	1.62	6.88×10^{-46}	6.88×10^{-45}
Unspecified asthma with (acute) exacerbation	J45.901	1.49	1.58	1.42	2.51×10^{-43}	2.76×10^{-42}
Epilepsy, unspecified, without mention of intractable epilepsy	345.9	1.86	2.03	1.71	2.08×10^{-41}	2.50×10^{-40}
Migraine, unspecified, without mention of intractable migraine, without mention of status migrainosus	346.9	1.53	1.63	1.45	5.85×10^{-41}	7.61×10^{-40}
CHLORIDE(CL)	CHLORIDE(CL)	1.41	1.49	1.35	1.58×10^{-36}	2.21×10^{-35}
Irritable bowel syndrome	564.1	1.89	2.09	1.72	5.50×10^{-32}	8.25×10^{-31}
RDW	RDW	1.52	1.63	1.42	2.38×10^{-30}	3.81×10^{-29}
Mononeuritis of unspecified site	355.9	1.45	1.54	1.37	5.31×10^{-30}	9.03×10^{-29}
Unspecified essential hypertension	401.9	1.73	1.89	1.58	2.36×10^{-29}	4.25×10^{-28}

Personal history of tobacco use	V15.82	1.33	1.40	1.27	4.27×10^{-28}	8.11×10^{-27}
Anemia, unspecified	285.9	1.44	1.53	1.36	1.15×10^{-27}	2.29×10^{-26}
MCH	MCH	1.50	1.61	1.40	3.55×10^{-27}	7.45×10^{-26}
Unspecified viral hepatitis C without hepatic coma	70.7	2.06	2.32	1.82	5.20×10^{-27}	1.14×10^{-25}
HGB	HGB	1.39	1.47	1.32	9.40×10^{-27}	2.16×10^{-25}
Supervision of normal first pregnancy	V22.0	0.460	0.523	0.402	9.56×10^{-27}	2.29×10^{-25}
HEMATOCRIT(HCT)	HEMATOCRIT(HCT)	1.38	1.46	1.31	2.54×10^{-26}	6.36×10^{-25}
Other specified disorders of stomach and duodenum	537.89	2.45	2.86	2.10	1.86×10^{-25}	4.83×10^{-24}
Osteoporosis, unspecified	733	2.45	2.88	2.09	5.31×10^{-24}	1.43×10^{-22}
Unspecified acquired hypothyroidism	244.9	1.47	1.57	1.37	5.65×10^{-24}	1.58×10^{-22}
Mononeuritis of unspecified site	355.9	1.90	2.13	1.69	6.43×10^{-24}	1.86×10^{-22}
MCV	MCV	1.50	1.61	1.39	1.52×10^{-23}	4.56×10^{-22}
Pure hyperglyceridemia	E78.1	1.35	1.43	1.28	1.65×10^{-23}	5.12×10^{-22}
WBC	WBC	1.37	1.45	1.29	9.61×10^{-23}	3.08×10^{-21}
Accident caused by hot liquids and vapors, including steam	E924.0	4.27	5.59	3.26	2.69×10^{-22}	8.88×10^{-21}
Chest pain, unspecified	R07.9	1.42	1.52	1.33	1.68×10^{-21}	5.71×10^{-20}
Accident caused by hot liquids and vapors, including steam	E924.0	4.13	5.43	3.14	1.63×10^{-20}	5.69×10^{-19}
Accident caused by hot liquids and vapors, including steam	E924.0	4.77	6.46	3.52	2.86×10^{-20}	1.03×10^{-18}
Osteoarthrosis, unspecified whether generalized or localized, site unspecified	715.9	1.41	1.51	1.32	1.89×10^{-19}	7.01×10^{-18}
Undiagnosed cardiac murmurs	785.2	1.80	2.03	1.60	5.08×10^{-19}	1.93×10^{-17}
ANION GAP	ANION GAP	1.44	1.55	1.34	1.45×10^{-18}	5.66×10^{-17}

BLOOD-URINE	BLOOD-URINE	1.29	1.35	1.22	1.51×10^{-18}	6.05×10^{-17}
Other and unspecified angina pectoris	413.9	2.46	2.97	2.04	6.49×10^{-18}	2.66×10^{-16}
ABS NEUTROPHILS	ABS NEUTROPHILS	1.29	1.36	1.22	1.48×10^{-17}	6.21×10^{-16}
Post-traumatic stress disorder, unspecified	F43.10	1.31	1.39	1.24	5.28×10^{-17}	2.27×10^{-15}
GLUCOSE-URINE	GLUCOSE-URINE	1.69	1.89	1.51	6.40×10^{-17}	2.82×10^{-15}
Personal history of allergy to other specified medicinal agents	V14.8	1.41	1.51	1.31	7.10×10^{-17}	3.19×10^{-15}
Long-term (current) use of steroids	V58.65	1.35	1.45	1.27	8.70×10^{-17}	4.00×10^{-15}
Personal history of malignant neoplasm of prostate	V10.46	1.49	1.62	1.36	3.34×10^{-16}	1.57×10^{-14}
Accident caused by other hot substance or object	E924.8	2.57	3.16	2.08	6.82×10^{-16}	3.27×10^{-14}
Accident caused by hot liquids and vapors, including steam	E924.0	3.49	4.61	2.64	1.09×10^{-15}	5.32× 10-14
Thalassemia, unspecified	282.4	8.26	13.32	5.12	3.35×10^{-15}	1.68×10^{-13}
Screening for malignant neoplasms of cervix	V76.2	0.849	0.881	0.818	4.31×10^{-15}	2.20×10^{-13}
Nausea with vomiting	787.01	1.36	1.46	1.27	4.56×10^{-15}	2.37×10^{-13}
Primary hypercoagulable state	289.81	2.80	3.54	2.21	4.72×10^{-15}	2.50×10^{-13}
Unspecified sleep apnea	780.57	1.32	1.40	1.24	4.85×10^{-15}	2.62×10^{-13}
GLUCOSE (BEDSIDE TEST)	GLUCOSE(BEDSIDE TEST)	1.38	1.49	1.28	6.23×10^{-15}	3.43×10^{-13}
Accident caused by hot liquids and vapors, including steam	E924.0	4.45	6.27	3.16	6.64×10^{-15}	3.72×10^{-13}
Acute sinusitis, unspecified	461.9	0.739	0.792	0.689	6.91×10^{-15}	3.94×10^{-13}
Arthropathy, unspecified, site unspecified	716.9	1.89	2.19	1.63	7.92×10^{-15}	4.60×10^{-13}
Screening for unspecified condition	V82.9	0.813	0.853	0.775	1.41×10^{-14}	8.30×10^{-13}
Activity, other specified	Y93.89	1.20	1.25	1.15	2.36× 10-14	1.42×10^{-12}

ABS BASOPHILS	ABS BASOPHILS	1.35	1.45	1.26	2.62×10^{-14}	1.60×10^{-12}
RBC	RBC	1.27	1.34	1.20	3.61×10^{-14}	2.24×10^{-12}
Unspecified transient cerebral ischemia	435.9	7.09	11.27	4.46	4.58×10^{-14}	2.88×10^{-12}
Other and unspecified rheumatic aortic diseases	395.9	8.08	13.29	4.91	6.53×10^{-14}	4.18×10^{-12}
POTASSIUM(K)	POTASSIUM(K)	1.28	1.35	1.20	7.41×10^{-14}	4.81×10^{-12}
Obesity, unspecified	E66.9	1.29	1.37	1.21	1.19×10^{-13}	7.98×10^{-12}
Osteitis deformans without mention of bone tumor	731	14.63	27.90	7.67	1.19×10^{-13}	7.99×10^{-12}
Malignant neoplasm of pancreas, part unspecified	157.9	0.131	0.214	0.080	1.29×10^{-13}	8.75×10^{-12}
VITAMIN D2 1.25 MG (50,000 UNIT)	DB00153|DB11094	0.673	0.740	0.611	1.33×10^{-13}	9.19×10^{-12}
Accident caused by other hot substance or object	E924.8	3.77	5.19	2.73	1.39×10^{-13}	9.72×10^{-12}
Pain in unspecified joint	M25.50	0.755	0.809	0.704	4.40×10^{-13}	3.12×10^{-11}
INR	INR	1.96	2.31	1.66	4.61×10^{-13}	3.32×10^{-11}
Accident caused by other hot substance or object	E924.8	3.00	3.95	2.28	1.17×10^{-12}	8.53×10^{-11}
Other convulsions	780.39	1.50	1.66	1.36	1.18×10^{-12}	8.77×10^{-11}
IONIZED CALCIUM, ISTAT	IONIZED CALCIUM, ISTAT	1.66	1.88	1.46	1.65×10^{-12}	1.24×10^{-10}
TACROLIMUS CAP 1MG	DB00864	0.415	0.520	0.332	3.63×10^{-12}	2.76×10^{-10}
Home accidents	E849.0	1.17	1.21	1.12	3.74×10^{-12}	2.88×10^{-10}
Other specified aftercare	V58.89	2.82	3.68	2.16	4.51×10^{-12}	3.52×10^{-10}
Chronic pancreatitis	577.1	2.25	2.78	1.82	8.20×10^{-12}	6.48×10^{-10}
IONIZED CALCIUM	IONIZED CALCIUM	0.498	0.598	0.415	9.33×10^{-12}	7.47×10^{-10}
Diverticulosis of colon (without mention of hemorrhage)	562.1	1.71	1.96	1.48	1.38×10^{-11}	1.12×10^{-09}
Acute, but ill-defined, cerebrovascular disease	436	4.09	5.93	2.82	1.71×10^{-11}	1.40×10^{-09}

Unspecified disorder of kidney and ureter	593.9	1.93	2.30	1.62	2.59×10^{-11}	2.15×10^{-09}
Accident caused by other hot substance or object	E924.8	2.97	3.97	2.22	2.92×10^{-11}	2.45×10^{-09}
MEPHYTON 5 MG TABLET	DB01022	0.248	0.361	0.171	3.39×10^{-11}	2.88×10^{-09}
Personal history of irradiation, presenting hazards to health	V15.3	0.641	0.723	0.568	7.04×10^{-11}	6.06×10^{-09}
BACTERIA	BACTERIA	1.23	1.30	1.16	9.21×10^{-11}	8.01×10^{-09}
Other specific disorders of sleep of nonorganic origin	307.49	0.563	0.659	0.481	1.02×10^{-10}	8.96×10^{-09}
Alzheimer's disease	331	3.10	4.23	2.27	1.32×10^{-10}	1.17× 10-08
Enlargement of lymph nodes	785.6	0.590	0.683	0.510	1.55×10^{-10}	1.40×10^{-08}
MYCOPHENOLATE 250 MG CAPSULES	DB00688	0.330	0.449	0.243	1.89×10^{-10}	1.72×10^{-08}
Unspecified hearing loss	389.9	1.85	2.19	1.56	2.45×10^{-10}	2.25×10^{-08}
Hyperosmolality and hypernatremia	E87.0	1.43	1.58	1.29	2.93×10^{-10}	2.72×10^{-08}
BASE EXCESS	BASE EXCESS	0.460	0.573	0.370	3.80×10^{-10}	3.57×10^{-08}
TOTAL PROTEIN	TOTAL PROTEIN	1.24	1.32	1.17	1.16×10^{-09}	1.10×10^{-07}
CALCIUM (CA)	CALCIUM(CA)	1.20	1.27	1.14	1.32×10^{-09}	1.27×10^{-07}
RED BLOOD CELLS-URINE	RED BLOOD CELLS-URINE	1.22	1.29	1.15	2.09×10^{-09}	2.03×10^{-07}
CARTIA XT 240 MG CAPSULE	DB00343	1.82	2.18	1.53	2.10×10^{-09}	2.06×10^{-07}
Other respiratory abnormalities	786.09	0.818	0.868	0.771	2.17×10^{-09}	2.15×10^{-07}
SODIUM (NA)	SODIUM(NA)	1.26	1.35	1.18	2.67×10^{-09}	2.67×10^{-07}
PROTHROMBIN TIME	PROTHROMBIN TIME	1.75	2.06	1.48	2.71×10^{-09}	2.74×10^{-07}
MPV	MPV	3.84	5.77	2.56	6.06×10^{-09}	6.19×10^{-07}
Atherosclerotic heart disease of native coronary artery without angina pectoris	I25.10	1.33	1.45	1.22	7.78×10^{-09}	8.01×10^{-07}

CEPHALEXIN 500 MG CAPSULE	DB00567	0.816	0.869	0.767	8.62×10^{-09}	8.97×10^{-07}
VOL-PLUS TAB	DB01592	0.794	0.852	0.739	9.33×10^{-09}	9.80×10^{-07}
STELARA 90 MG/ML SYRINGE	DB05679	3.05	4.30	2.17	1.02×10^{-08}	1.08×10^{-06}
PLATELETS	PLATELETS	1.29	1.39	1.19	1.03×10^{-08}	1.10×10^{-06}
Anorexia nervosa	307.1	2.50	3.31	1.88	1.30×10^{-08}	1.41×10^{-06}
Gastroschisis	756.73	4.08	6.30	2.64	1.33×10^{-08}	1.44×10^{-06}
Complications of transplanted lung	996.84	0.273	0.408	0.183	1.43×10^{-08}	1.57×10^{-06}
MONUROL 3 GM SACHET	DB00828|DB03754	0.344	0.479	0.247	1.78×10^{-08}	1.98×10^{-06}
Liver replaced by transplant	V42.7	1.97	2.44	1.60	1.99×10^{-08}	2.23×10^{-06}
CHOLESTEROL	CHOLESTEROL	0.828	0.878	0.780	2.35×10^{-08}	2.66×10^{-06}
ENALAPRIL 20 MG TABLETS	DB00584	0.375	0.511	0.276	2.42×10^{-08}	2.76×10^{-06}
NEUT	NEUT	0.530	0.647	0.434	2.50×10^{-08}	2.88×10^{-06}
TROSPIUM CHLORIDE ER 60 MG CAP	DB00209	2.25	2.91	1.74	2.70×10^{-08}	3.13×10^{-06}
VITAMIN B12 500 MCG TABLET	DB00115	0.755	0.826	0.690	4.03×10^{-08}	4.71×10^{-06}
LYMPH	LYMPH	0.544	0.662	0.447	5.77×10^{-08}	6.80×10^{-06}
ASPARTATE AMINOT (AST)	ASPARTATE AMINOT.(AST)	1.17	1.24	1.11	6.09×10^{-08}	7.25×10^{-06}
SAW PALMETTO 160 MG CAPSULE	DB14360	0.403	0.540	0.300	6.10×10^{-08}	7.32×10^{-06}
LIPASE	LIPASE	1.31	1.43	1.20	6.34×10^{-08}	7.67×10^{-06}
Acute bronchitis	466	0.794	0.856	0.737	6.92×10^{-08}	8.45×10^{-06}
Chronic gastric ulcer without mention of hemorrhage or perforation, without mention of obstruction	531.7	2.86	4.02	2.03	7.16×10^{-08}	8.80×10^{-06}
Other musculoskeletal symptoms referable to limbs	729.89	0.526	0.648	0.426	8.00×10^{-08}	9.92×10^{-06}

Paralysis agitans	332	3.31	4.88	2.24	8.55× 10^{-08}	1.07× 10^{-05}
Mitral valve insufficiency and aortic valve insufficiency	396.3	1.80	2.18	1.49	8.67× 10^{-08}	1.09× 10^{-05}
Enthesopathy of hip region	726.5	0.435	0.571	0.331	8.76× 10^{-08}	1.11× 10^{-05}
Carcinoma in situ of anal canal	230.5	0.407	0.546	0.303	9.52× 10^{-08}	1.22× 10^{-05}
IGA	IGA	2.23	2.90	1.71	1.08× 10^{-07}	1.40× 10^{-05}
LEUKOCYTE ESTERASE	LEUKOCYTE ESTERASE	1.18	1.24	1.11	1.26× 10^{-07}	1.64× 10^{-05}
EPITHELIAL CELLS	EPITHELIAL CELLS	1.21	1.29	1.14	1.40× 10^{-07}	1.84× 10^{-05}
MYCOPHENOLIC ACID DR 180 MG TB	DB01024	0.295	0.443	0.197	1.46× 10^{-07}	1.93× 10^{-05}
PREDNISONE TAB 10 MG	DB00635	0.835	0.886	0.786	1.60× 10^{-07}	2.12× 10^{-05}
Pneumonia, organism unspecified	486	0.759	0.832	0.692	1.80× 10^{-07}	2.41× 10^{-05}
Chronic airway obstruction, not elsewhere classified	496	1.26	1.36	1.17	1.87× 10^{-07}	2.52× 10^{-05}
Impetigo	684	2.78	3.92	1.97	1.87× 10^{-07}	2.54× 10^{-05}
Mononeuritis of unspecified site	355.9	1.78	2.16	1.47	1.87× 10^{-07}	2.56× 10^{-05}
Mild dysplasia of cervix	622.11	1.85	2.27	1.50	1.94× 10^{-07}	2.67× 10^{-05}
SUPREP BOWEL PREP KIT	DB14500	0.848	0.897	0.802	2.61× 10^{-07}	3.63× 10^{-05}
Disturbance of skin sensation	782	0.799	0.862	0.740	2.80× 10^{-07}	3.92× 10^{-05}
Acquired absence of intestine (large) (small)	V45.72	1.28	1.40	1.18	3.33× 10^{-07}	4.70× 10^{-05}
Open wound of knee, leg (except thigh), and ankle, without mention of complication	891	0.608	0.721	0.513	3.95× 10^{-07}	5.61× 10^{-05}
KETONES-URINE	KETONES-URINE	1.19	1.27	1.12	4.46× 10^{-07}	6.37× 10^{-05}
MCHC	MCHC	1.20	1.27	1.12	4.67× 10^{-07}	6.73× 10^{-05}
WHITE BLOOD CELLS-URINE	WHITE BLOOD	1.21	1.30	1.13	5.95× 10^{-07}	8.62× 10-05

	CELLS-URINE					
Unspecified immunity deficiency	279.3	3.41	5.24	2.22	6.54×10^{-07}	9.55×10^{-05}
Abnormal cardiovascular function study, unspecified	794.3	0.776	0.848	0.709	7.46×10^{-07}	0.000110
Poisoning by unspecified drug or medicinal substance	977.9	1.42	1.62	1.26	1.12×10^{-06}	0.000166
ZENATANE 20 MG CAPSULE	DB00982	3.77	6.06	2.35	1.24×10^{-06}	0.000185
Other nonspecific findings on examination of urine	791.9	0.660	0.766	0.569	1.30×10^{-06}	0.000195
Sprains and strains of unspecified site of knee and leg	844.9	0.468	0.617	0.355	2.06×10^{-06}	0.000311
TOBRAMYCIN-DEXAMETH OPHTH SUSP	DB00684|DB01234	0.665	0.771	0.573	2.06×10^{-06}	0.000314
Malignant neoplasm of kidney, except pelvis	189	0.361	0.524	0.249	2.27×10^{-06}	0.000347
TORSEMIDE 10 MG TABLETS	DB00214	3.71	5.98	2.30	2.34×10^{-06}	0.000360
Supervision of other high-risk pregnancy	V23.89	0.678	0.782	0.588	2.42×10^{-06}	0.000375
Other emphysema	492.8	0.656	0.766	0.562	2.48×10^{-06}	0.000388
Colostomy status	V44.3	2.00	2.59	1.55	2.70×10^{-06}	0.000423
Personal history of noncompliance with medical treatment, presenting hazards to health	V15.81	1.22	1.32	1.14	2.84×10^{-06}	0.000448
VITAMIN B12	VITAMIN B12	0.800	0.869	0.737	3.24×10^{-06}	0.000515
Teething syndrome	520.7	0.528	0.670	0.416	3.81×10^{-06}	0.000609
Unspecified polyarthropathy or polyarthritis, site unspecified	716.5	0.408	0.570	0.292	3.82×10^{-06}	0.000615
Malignant neoplasm of larynx, unspecified	161.9	0.274	0.445	0.168	4.18×10^{-06}	0.000676
UROBILINOGEN-URINE	UROBILINOGEN-URINE	0.823	0.885	0.765	4.18×10^{-06}	0.000681

Other B-complex deficiencies	266.2	0.664	0.774	0.569	4.38×10^{-06}	0.000719
Rosuvastatin 10 Mg Tab Apot	DB01098	1.71	2.10	1.40	4.42×10^{-06}	0.000730
Other specified anomalies of stomach	750.7	0.529	0.672	0.416	4.47×10^{-06}	0.000742
Occlusion and stenosis of carotid artery without mention of cerebral infarction	433.1	0.504	0.652	0.389	4.70×10^{-06}	0.000785
Paroxysmal supraventricular tachycardia	427	0.542	0.683	0.430	4.84×10^{-06}	0.000813
Other, mixed, or unspecified drug abuse, unspecified	305.9	1.617	1.938	1.348	5.11×10^{-06}	0.000864
VERY LOW DENSITY LIPOPROTEIN	VERY LOW DENSITY LIPOPROTEIN	0.765	0.847	0.692	5.37×10^{-06}	0.000913
DULOXETINE HCL DR 30 MG CAP	DB00476	1.26	1.37	1.15	5.45×10^{-06}	0.000932
TRILYTE SOL	DB01390|DB09153	0.678	0.786	0.585	6.08×10^{-06}	0.00105
Other, mixed, or unspecified drug abuse, unspecified	305.9	1.36	1.52	1.21	6.75×10^{-06}	0.00117
Phlebitis and thrombophlebitis of deep veins of lower extremities, other	451.19	5.20	9.76	2.77	6.75×10^{-06}	0.00117
Chronic mycotic otitis externa	380.15	0.582	0.716	0.473	6.87×10^{-06}	0.00120
TRICYCLIC ANTIDEPRESSANTS	TRICYCLIC ANTIDEPRESSANTS	1.51	1.76	1.29	6.95×10^{-06}	0.00122
PROTEIN-URINE	PROTEIN-URINE	1.16	1.23	1.10	7.03×10^{-06}	0.00124
PREZISTA 800 MG TABLET	DB00898|DB01264	4.77	8.68	2.62	7.09×10^{-06}	0.00126
Proteinuria	791	0.466	0.625	0.347	7.75×10^{-06}	0.00139
Unspecified vitamin D deficiency	268.9	0.822	0.887	0.762	1.01×10^{-05}	0.0018
CARBON DIOXIDE (CO2)	CARBON DIOXIDE(CO2)	1.18	1.26	1.11	1.02×10^{-05}	0.00184

Other persistent mental disorders due to conditions classified elsewhere	294.8	0.452	0.618	0.330	1.41×10^{-05}	0.00256
Congestive heart failure, unspecified	428	1.39	1.58	1.22	1.42×10^{-05}	0.00260
Temporomandibular joint disorders, unspecified	524.6	0.668	0.784	0.570	1.42×10^{-05}	0.00260
Alexia and dyslexia	784.61	0.288	0.471	0.176	1.43×10^{-05}	0.00265
PRE ALBUMIN	PRE ALBUMIN	1.63	1.98	1.34	1.44×10^{-05}	0.00267
Mitral valve disorders	424	1.61	1.94	1.33	1.55×10^{-05}	0.00289
UREA NITROGEN	UREA NITROGEN	1.19	1.27	1.11	1.55×10^{-05}	0.00292
DIPHENOXYLATE ATROPINE 2 5	DB00572 | DB01081	0.537	0.688	0.420	1.61×10^{-05}	0.00305
Ganglion of joint	727.41	0.518	0.673	0.399	1.66×10^{-05}	0.00316
ETHANOL (SERUM/PLASMA)	ETHANOL (SERUM/PLASMA)	1.33	1.50	1.19	1.73×10^{-05}	0.00330
Other complications due to genitourinary device, implant, and graft	996.76	0.661	0.780	0.561	1.74×10^{-05}	0.00335
Atrophic gastritis, without mention of hemorrhage	535.1	1.23	1.34	1.13	2.07×10^{-05}	0.00399
Drug withdrawal	292	1.92	2.50	1.48	2.15×10^{-05}	0.00417
DISULFIRAM 250 MG TABLET	DB00822	0.317	0.504	0.199	2.27×10^{-05}	0.00442
Disability examination	V68.01	0.707	0.814	0.614	2.53×10^{-05}	0.00495
Chronic hepatitis C without mention of hepatic coma	70.54	1.54	1.84	1.29	2.57×10^{-05}	0.00508
Malignant neoplasm of bone and articular cartilage, site unspecified	170.9	1.97	2.59	1.49	2.57×10^{-05}	0.00510
UIBC	UIBC	0.392	0.575	0.268	2.72×10^{-05}	0.00542
AMOXICILLIN CAP 500 MG	DB01060	0.894	0.936	0.854	2.87×10^{-05}	0.00574
Submucous leiomyoma of uterus	218	0.662	0.784	0.559	3.22×10^{-05}	0.00650
Issue of repeat prescriptions	V68.1	1.52	1.81	1.28	3.22×10^{-05}	0.00651

DEXILANT DR 60 MG CAPSULE	DB05351	0.573	0.721	0.456	3.29×10^{-05}	0.00667
Rheumatoid arthritis	714	1.60	1.94	1.32	3.64×10^{-05}	0.00742
Myalgia and myositis, unspecified	729.1	1.48	1.74	1.26	3.78×10^{-05}	0.00774
VESICARE TAB 5 MG	DB01591	1.86	2.41	1.44	3.80×10^{-05}	0.00784
Muscle spasm of back	M62.830	0.729	0.831	0.639	3.90×10^{-05}	0.00807
Unspecified ectopic pregnancy without intrauterine pregnancy	633.9	0.277	0.473	0.163	4.07×10^{-05}	0.00847
Cystic fibrosis with pulmonary manifestations	277.02	0.199	0.391	0.102	4.45×10^{-05}	0.00930
LOW DENSITY LIPOPROTEIN	LOW DENSITY LIPOPROTEI N	0.834	0.900	0.773	4.68×10^{-05}	0.00983
TRIAMCINOLON OIN 0.1%	DB00620	0.804	0.882	0.734	5.33×10^{-05}	0.0113
ACARBOSE 25 MG TABLET	DB00284	2.03	2.74	1.51	5.33×10^{-05}	0.0113
Malignant neoplasm of tonsil	146	0.420	0.605	0.291	5.33×10^{-05}	0.0114
ALPRAZOLAM 0.5 MG TABLET	DB00404	1.34	1.51	1.18	5.34×10^{-05}	0.0114
Influenza with other respiratory manifestations	487.1	0.665	0.790	0.560	5.43×10^{-05}	0.0117
Sprains and strains of other specified sites of shoulder and upper arm	840.8	0.727	0.831	0.635	5.43×10^{-05}	0.0117
Gout, unspecified	274.9	1.79	2.28	1.40	5.48×10^{-05}	0.0119
EVEROLIMUS	EVEROLIMU S	3.62	6.23	2.10	5.48×10^{-05}	0.0119
Cocaine abuse, unspecified	305.6	1.46	1.71	1.24	5.85×10^{-05}	0.0128
Other venous complications of pregnancy and the puerperium, delivered, with or without mention of antepartum condition	671.81	0.467	0.646	0.338	6.21×10^{-05}	0.0137
ZOLPIDEM TARTRATE 10 MG TABLET	DB00425	0.717	0.826	0.622	6.75×10^{-05}	0.0149

Hypertonicity of bladder	596.51	0.651	0.782	0.542	6.77×10^{-05}	0.0150
CREATININE	CREATININE	1.21	1.32	1.12	7.04×10^{-05}	0.0157
MY WAY 1.5 MG TABLET	DB00367	0.749	0.848	0.662	7.11×10^{-05}	0.0159
QUINAPRIL 40 MG TABLET	DB00881	3.17	5.19	1.93	7.15×10^{-05}	0.0161
CYCLOSPORIN MONOCLONAL ASSAY	CYCLOSPORIN MONOCLONAL ASSAY	0.378	0.573	0.249	7.15×10^{-05}	0.0162
Chronic laryngitis	476	0.248	0.452	0.136	7.62×10^{-05}	0.0173
Twin pregnancy, antepartum condition or complication	651.03	0.146	0.335	0.064	7.92×10^{-05}	0.0180
DICYCLOMINE 10 MG CAPSULE	DB00804	0.768	0.861	0.686	8.20×10^{-05}	0.0188
Malignant neoplasm of liver, primary	155	3.34	5.63	1.98	9.09×10^{-05}	0.0209
Fibromyalgia	M79.7	1.15	1.23	1.08	9.93×10^{-05}	0.0229
Thyrotoxicosis without mention of goiter or other cause, and without mention of thyrotoxic crisis or storm	242.9	0.554	0.717	0.427	0.000104	0.0240
Myrbetriq Tab 25 mg	DB08893	1.97	2.65	1.46	0.000106	0.0247
Melanoma of skin, site unspecified	172.9	0.297	0.506	0.175	0.000109	0.0255
Rheumatic tricuspid insufficiency	I07.1	0.664	0.795	0.555	0.000109	0.0257
PERMETHRIN CRE 5%	DB04930	0.680	0.805	0.574	0.000109	0.0257
Elevated prostate specific antigen (PSA)	790.93	0.421	0.616	0.288	0.00011	0.0261
Hip joint replacement	V43.64	0.725	0.835	0.630	0.000114	0.0271
BANDS %	BANDS %	0.575	0.734	0.451	0.000119	0.0283
ALMOTRIPTAN MALATE 12.5 MG TAB	DB00918	2.69	4.16	1.73	0.000128	0.0306
TUMOR HCG & QUANT. PREGNANCY	TUMOR HCG & QUANT. PREGNANCY	0.697	0.818	0.594	0.000136	0.0328

FARXIGA 5 MG TABLET	DB06292	0.567	0.730	0.440	0.000 142	0.0343
PROTEIN - URINE (POC)	PROTEIN - URINE (POC)	0.835	0.905	0.771	0.000 147	0.0356
Alcoholic liver damage, unspecified	571.3	0.513	0.691	0.381	0.000 148	0.0362
Juvenile osteochondrosis of lower extremity, excluding foot	732.4	0.300	0.514	0.175	0.000 151	0.0370
ABS MONOCYTES	ABS MONOCYTES	1.16	1.24	1.08	0.000 151	0.0371
LUPRON DEPOT 11.25 MG 3MO KIT	DB00007	0.361	0.570	0.229	0.000 153	0.0378
EOSINOPHIL	EOSINOPHIL	0.688	0.814	0.582	0.000 155	0.0386
Alcohol abuse, unspecified	305	1.43	1.68	1.22	0.000 161	0.0400
ALANINE AMINOTRANS(ALT)	ALANINE AMINOTRAN S(ALT)	1.14	1.21	1.08	0.000 162	0.0405
Urinary frequency	788.41	0.818	0.896	0.748	0.000 167	0.0420
Late effects of injury purposely inflicted by other person	E969	1.85	2.44	1.40	0.000 167	0.0422
Other specified disorders of rectum and anus	569.49	1.37	1.58	1.19	0.000 176	0.0445
Atony of bladder	596.4	1.89	2.51	1.42	0.000 178	0.0451
INGREZZA CAP 40MG	DB11915	1.91	2.55	1.42	0.000 185	0.0471

Notes: RDW: Red cell distribution width, MCH: Mean corpuscular hemoglobin, HGB: Hemoglobin, HCT: Hematocrit, MCV: Mean corpuscular volume, WBC: White blood cell, ABS Neutrophils: Absolute neutrophil count, ABS Basophils: Absolute basophil count, RBC: Red blood cell, INR: International normalized ratio, Ionized calcium ISTAT: Ionized calcium instrument description and testing technologies, MPV: Mean platelet volume. Relative contribution value > 1: Risk, Relative contribution value < 1: Protective, FDR_Q: false discovery rate adjusted Q value. The diagnostic codes mentioned in the table above (Diagnosis code/DrugBankID/lab tests) and used in our study are representatives of all 1639 diagnosis groups.

Table S2: Performance of traditional machine learning algorithms

	**DT**	**RF**	**LR**	**RF_validation**	**DT_validation**	**LR_validation**
TP	585	454	676	440	436	636
FP	200	70	170	59	201	187
TN	875	1005	905	996	854	868
FN	490	621	399	615	619	419
TPR	0.544	0.422	0.629	0.417	0.413	0.603
PPV	0.745	0.866	0.799	0.882	0.684	0.773
NPV	0.641	0.618	0.694	0.618	0.580	0.674
F1	0.629	0.568	0.704	0.566	0.515	0.677
AUC	0.679	0.679	0.735	0.681	0.611	0.713

Notes: All models were set at a random state of 42 to ensure reproducibility, while the other hyper-parameters were left at default settings. The random state seeded the random number generator used in the models. For the final random forest model, we set estimators to 100 and the maximum number of features to the square root of the number of features. Because these two machine learning approaches cannot consider time, we only used the indicators as input. That is, if any abnormal lab test was reported within 1 year preceding the index date, that indicator was set as 1, otherwise as 0. TP: true positives; TN: true negatives; FP: false positives; FN: false negatives; TPR: true positive rate; PPV: positive predictive value; NPV: negative predictive value; AUC: area under curve; DT: decision tree; RF: random forest; LR: logistic regression.
